# γ‐Secretase modulators show selectivity for γ‐secretase–mediated amyloid precursor protein intramembrane processing

**DOI:** 10.1111/jcmm.17146

**Published:** 2021-12-20

**Authors:** Tobias A. Weber, Johan Lundkvist, Johanna Wanngren, Hlin Kvartsberg, ShaoBo Jin, Pia Larssen, Dan Wu, Daniel V. Oliveira, Karolina Minta, Gunnar Brinkmalm, Henrik Zetterberg, Kaj Blennow, Gunnar Nordvall, Bengt Winblad, Erik Portelius, Helena Karlström

**Affiliations:** ^1^ Division of Neurogeriatrics Department of Neurobiology, Care Science and Society Karolinska Institutet Stockholm Sweden; ^2^ Medical Scientific Affairs Aesculap AG Tuttlingen Germany; ^3^ Alzecure Pharma Huddinge Sweden; ^4^ Sinfonia Biotherapeutics AB Huddinge Sweden; ^5^ Department of Psychiatry and Neurochemistry Institute of Neuroscience and Physiology The Sahlgrenska Academy at the University of Gothenburg Gothenburg Sweden; ^6^ Clinical Neurochemistry Laboratory Sahlgrenska University Hospital Mölndal Sweden; ^7^ Department of Cell and Molecular Biology Karolinska Institutet Stockholm Sweden; ^8^ Department of Obstetrics and Gynecology First Affiliated Hospital of Nanjing Medical University Nanjing China; ^9^ Department of Neurodegenerative Disease UCL Institute of Neurology London UK; ^10^ UK Dementia Research Institute at UCL London UK; ^11^ Theme Aging Geriatric Clinic Karolinska University Hospital Huddinge Sweden

**Keywords:** beta‐amyloid, gamma‐secretase modulators, presenilin, substrates

## Abstract

The aggregation of β‐amyloid peptide 42 results in the formation of toxic oligomers and plaques, which plays a pivotal role in Alzheimer's disease pathogenesis. Aβ42 is one of several Aβ peptides, all of Aβ30 to Aβ43 that are produced as a result of γ‐secretase–mediated regulated intramembrane proteolysis of the amyloid precursor protein. γ‐Secretase modulators (GSMs) represent a promising class of Aβ42‐lowering anti‐amyloidogenic compounds for the treatment of AD. Gamma‐secretase modulators change the relative proportion of secreted Aβ peptides, while sparing the γ‐secretase–mediated processing event resulting in the release of the cytoplasmic APP intracellular domain. In this study, we have characterized how GSMs affect the γ‐secretase cleavage of three γ‐secretase substrates, E‐cadherin, ephrin type A receptor 4 (EphA4) and ephrin type B receptor 2 (EphB2), which all are implicated in important contexts of cell signalling. By using a reporter gene assay, we demonstrate that the γ‐secretase–dependent generation of EphA4 and EphB2 intracellular domains is unaffected by GSMs. We also show that γ‐secretase processing of EphA4 and EphB2 results in the release of several Aβ‐like peptides, but that only the production of Aβ‐like proteins from EphA4 is modulated by GSMs, but with an order of magnitude lower potency as compared to Aβ modulation. Collectively, these results suggest that GSMs are selective for γ‐secretase–mediated Aβ production.

## 1 INTRODUCTION

Alzheimer's disease (AD) is the most common form of dementia among the elderly and affects approximately 40 million people worldwide. The β‐amyloid peptide 42 (Aβ42) is the principal component of amyloid plaques that are one of the neuropathological hallmarks of AD. A large body of evidence, including human genetics, suggests that the deposition of Αβ42 is a key pathogenic event in AD,^1^ and recently, the monoclonal antibody aducanumab was approved as the first Aβ‐amyloid–directed therapeutic for the treatment of AD.

Aβ‐peptides are generated from the amyloid precursor protein (APP) via the sequential processing by β‐ and γ‐secretase.^2^ β‐Secretase cleavage results in shedding of most of the APP ectodomain, a peptide called sAPPβ, and leaves a 99 amino acid C‐terminal membrane integral fragment, C99, that retains 28 aa of the extracellular domain (EC), the transmembrane region (TMD) and a 50 amino acid intracellular domain (ICD). The short ectodomain of C99 is a critical substrate recognition motif for γ‐secretase and enables the recruitment of the substrate into the active site and subsequent proteolysis of the TMD. This mechanism has been denoted regulated intramembrane proteolysis (RIP).^3^ Several Aβ peptides of various lengths are generated as the result of γ‐secretase–mediated RIP.^4^ Initially, γ‐secretase cleaves C99 close to the inner leaflet of the membrane, the ‘ε‐site’. This cleavage results in the liberation of the APP intracellular domain (AICD), and a N‐terminal 50–51 amino acid long membrane retained Aβ peptide. The latter peptide is then subjected to further γ‐secretase–catalysed carboxy‐endoproteolysis in a continuous precursor‐product manner, which result in the production and release of shorter Aβ peptides that vary from 30 to 43 amino acids in length (i.e., Aβ30 to Aβ43).^5^, ^6^, ^7^, ^8^, ^9^ This mechanism of sequential γ‐secretase–mediated processing may not be unique to the proteolysis of APP processing since several Aβ‐like peptides have been detected in experiments studying the processing of other substrates.^10^, ^11^, ^12^

Both β‐ and γ‐secretase are appealing drug targets for preventing Aβ‐amyloidosis. However, there exist a large number of substrates for both enzymes that are involved in many important signalling cascades and pivotal physiological functions. There is therefore a potential risk for safety liabilities in response to enzyme inhibition. Indeed, several γ‐β‐secretase inhibitors have been precluded from further clinical development due to severe side effects, such as worsening of cognitive decline, gastrointestinal problems and an increased incidence of cancer.^13^, ^14^ Clearly, these problems with enzyme inhibitors call for alternative, more tolerable treatments to halt amyloidogenic Aβ production.^13^

In 2003, Koo and colleagues demonstrated that some nonsteroidal anti‐inflammatory drugs (NSAIDs) could modulate the Aβ profile in cell culture and in vivo, while γ‐secretase–mediated Notch signalling appeared spared.^15^, ^16^ The NSAIDs were found to decrease Aβ42 and Aβ40 secretion accompanied by a dose‐dependent increase in the less amyloidogenic Aβ38 and Aβ37 peptides. As a result, the total amount of secreted Aβ was not affected. These molecules and more recent discovered compounds exhibiting improved potency^17^, ^18^, ^19^ were denoted as γ‐secretase modulators (GSM), since they were shown to act at the level of γ‐secretase but without affecting the overall rate of APP nor Notch processing.

The mechanism of action of GSMs is still not fully understood, but recent scientific progress has identified an allosteric GSM binding site in presenilin (PS), the catalytic subunit of γ‐secretase.^20^ In terms of APP processing, the Aβ modulatory effects appeared to be caused by a GSM‐induced change in the cleavage site preference of γ‐secretase along the APP intramembranous domain.^5^, ^21^, ^22^ While the pharmacology of GSMs has been thoroughly studied regarding APP and Notch processing, less is known about other γ‐secretase reactions. Intriguingly, we and others have demonstrated that γ‐secretase–mediated processing of Notch appears to be less sensitive to GSMs as compared to APP processing, suggesting^5^, ^10^, ^11^, ^23^, ^24^ that it is possible to generate substrate‐selective GSMs.^10^, ^11^, ^23^ Studying γ‐secretase–mediated intramembrane processing beyond APP processing and Aβ peptide generation is complicated by the general lack of antibodies to delineate specific post‐proteolytic Aβ‐like peptides. In order to solve this issue, we have developed an experimental approach where we have fused a FLAG‐tag to the N‐terminus of the extracellular juxtamembrane region of Notch, allowing anti‐FLAG–directed immunoprecipitation and mass spectrometric analysis of secreted FLAG‐Nβ peptides.^10^, ^11^ This approach enabled us to monitor a number of FLAG‐Nβ peptides that correlated with Nβ peptides generated in other experimental systems devoid of a FLAG‐tag. These findings suggest that the presence of the N‐terminal FLAG‐tag does not affect γ‐secretase processing of Notch.^5^, ^10^, ^11^, ^17^, ^23^, ^25^, ^26^

In this study, we have taken advantage of the N‐terminal FLAG‐tagging strategy to extend our analysis of the pharmacology of GSM to less well‐characterized γ‐secretase–catalysed reactions. We have focused our efforts on exploring whether GSMs affect the processing of E‐cadherin, ephrin type B receptor 2 (EphB2) and ephrin type A receptor 4 (EphA4), which are known to mediate important cell signalling and may have been associated with severe side effects in response to γ‐secretase inhibitor trials (i.e., semagacestat and avagacestat).^27^, ^28^, ^29^, ^30^, ^31^, ^32^, ^33^, ^34^ We find that GSMs, in contrast to their effect on APP processing, do not affect the processing of E‐cadherin, EphB2 and EphA4 to a large extent. Rather, our data suggest that GSMs are selective for γ‐secretase–mediated APP processing and support the further development of GSMs as a tolerable and effective anti‐amyloidogenic treatment for the treatment of AD.

## 2 MATERIALS AND METHODS

### 2.1 Compounds

The GSIs L685,458 and DAPT were obtained from Sigma‐Aldrich, and Semagacestat was obtained from Divbio Science. AZ4126, AZ4800 and E2012 were prepared as previously described,^17^ according to patent numbers WO2010132015, WO2010053438 and WO2007139149.

### 2.2 cDNA constructs

The generation of the constructs and oligonucleotides used is described in Tables S1 and S2

### 2.3 Cell culture

HEK293 cells stably expressing human FLAG‐EphA4‐ΔE (FLAG‐A4ΔE), FLAG‐EphB2‐ΔE (FLAG‐B2ΔE), FLAG‐E‐cadherin‐ΔE (FLAG‐CadΔE) and FLAG‐C55 were cultured in Dulbecco´s modified Eagle's medium supplemented with 10% FBS and 400 µg/ml G418 or 200 µg/ml hygromycin. The APPswe‐overexpressing HEK293 cells were described previously.^10^ For each experiment, cells were plated the day before treatment. The cells were then treated with either L685,458 (1 µM), DAPT (10 µM), Semagacestat (1 µM), GSM AZ4126 (0.1, 0.3, 0.4 or 1 µm), GSM AZ4800 (400 nM), GSM E2012 (1 µm) or vehicle control (dimethyl sulphoxide (DMSO)).

### 2.4 Immunoblotting

Cells were treated as described above and lysed in cell lysis buffer (10 mM Tris, pH 8.1, 1 mM EDTA, 150 mM NaCl, 0.65% IGEPAL CA‐630) supplemented with cOmplete™ protease inhibitor cocktail (Roche). Protein levels were determined by the BCA protein assay kit (Pierce) and subjected to SDS‐PAGE and immunoblotting using the following antibodies: α‐VP16 (Abcam, ab4808), α‐FLAG (F3165, Sigma‐Aldrich), α‐AICD (Y188, Abcam, ab32136), α‐β‐tubulin (Abcam, ab179513) and appropriate HRP‐conjugated secondary antibodies. The blots were developed using Amersham Biosciences HyperfilmTM ECL (GE Healthcare) or the CCD camera LAS‐3000 (FUJIFILM Life Science).

### 2.5 Immunoprecipitation (IP) and mass spectrometry (MS) analysis

Aβ, FLAG‐Aβ and FLAG‐Aβ‐like peptides in media from FLAG‐EphA4‐ΔE–, FLAG‐EphB2‐ΔE–, FLAG‐CadΔE–, FLAG‐C55– and APPswe‐expressing HEK293 cells were immunoprecipitated with 40 µl α‐Flag M2 magnetic beads (Sigma‐Aldrich) or the anti‐Aβ–directed 4G8 antibody. The samples were incubated overnight at 4°C, and washed three times in phosphate‐buffered saline (PBS, pH 7.4) and one time in 50mM ammonium bicarbonate. The immunoprecipitated peptides were eluted in 100 µl 0.5% formic acid (FA), dried in a vacuum centrifuge, redissolved in 5 µl 0.1% FA in 20% acetonitrile and subsequently analysed using a Bruker Daltonics UltraFleXtreme matrix‐assisted laser desorption/ionization time‐of‐flight/time‐of‐flight (MALDI‐TOF/TOF) MS (Bruker Daltonics) as described elsewhere.^35^ Experiments were performed in triplicate and repeated three times (in the case of E‐cadherin, twice). The peak intensities were normalized to the sum of the peak intensities of all peptides in the spectra, and average was made from triplicated samples. All reported m/z are the monoisotopic peak of the protonated molecule [M+ H]+, and a mass deviation ≤50 ppm ((mass measured – theoretical mass) / theoretical mass) was considered as a match. Each spectrum was manually inspected.

### 2.6 Luciferase‐based Reporter Gene Assay

The luciferase‐based reporter gene assay was carried out as described previously^36^, ^37^ with slight modifications. In brief, HEK293 or PS‐deficient BD8 cells were transfected (Lipofectamine 2000, Invitrogen) with expression vectors encoding: C99‐GVP, E‐cadherin‐GVPΔE, EphA4‐GVPΔE and EphB2‐GVPΔE together with the plasmids MH100 and CMV‐β‐gal. In some experiments, BD8 cells were transfected with either a PS1‐ or a PS2‐encoding plasmid. Cells were then lysed 24 h post‐transfection in lysis buffer (see above), and luciferase and β‐galactosidase activities were measured as previously described. The experiments were performed in triplicates and repeated three times.

### 2.7 Quantification of Secreted Aβ from Cells

FLAG‐C55–expressing cells were exposed to GSIs, GSMs or vehicle control for 15 h, and the conditioned media were then analysed for Aβ 38, 40 and 42 using the Mesoscale Discovery Aβ‐triplex Assay. Aβ37 was monitored with an in‐house‐made ELISA, as described previously.^5^

### 2.8 Statistical analysis

Multiple comparisons were evaluated by one‐way ANOVA using GraphPad Prism 9, and values are mean +/− SEM. Student's *t* test was used to assess the statistical differences between two experimental groups.

## 3 RESULTS

### 3.1 GSMs do not affect the processing and intracellular domain formation of E‐cadherin, EphB2 and EphA4

**FIGURE 1:**
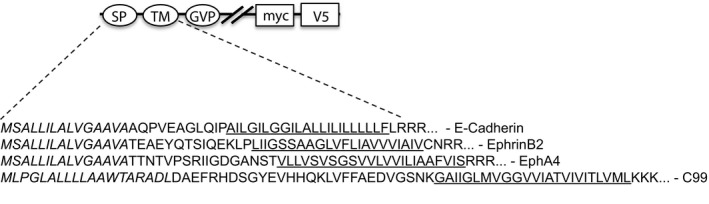
Schematic display of the ectodomain‐shedded γ‐secretase substrates. E‐cadherin, EphB2, EphA4 and C99 substrates with inserted Gal4‐VP16 domain (GVP). The signal peptide is depicted in italics, and the transmembrane domain (TMD) is underlined, myc: myc‐tag, V5: V5‐tag

**FIGURE 2:**
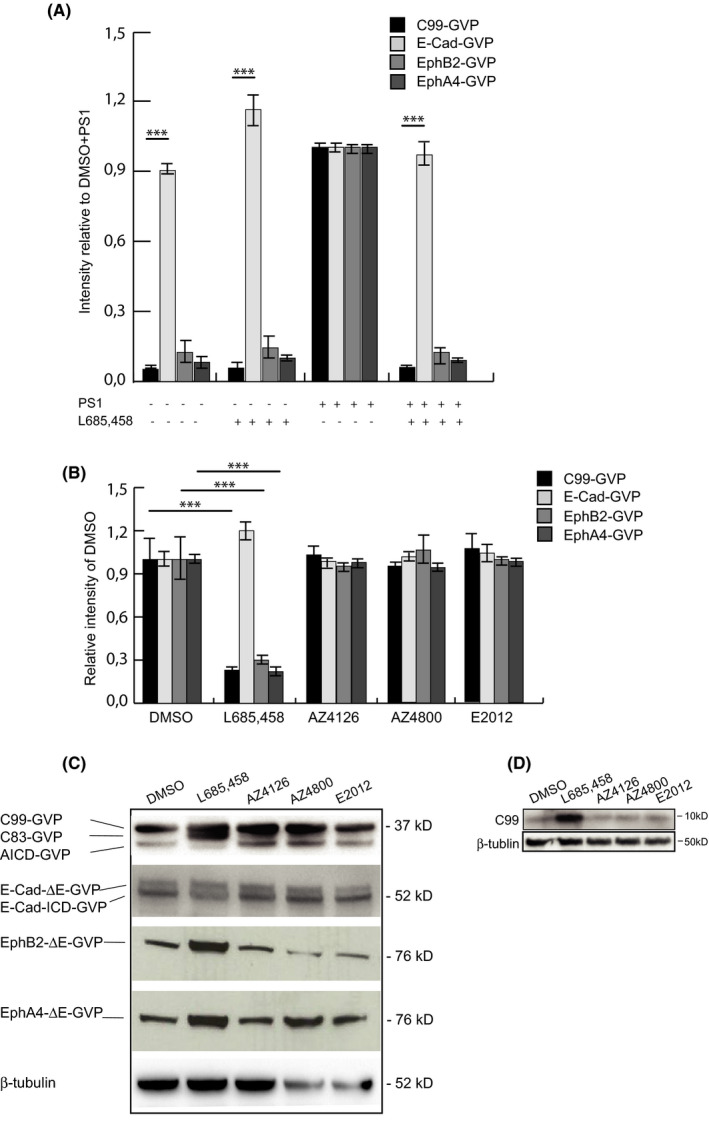
GSMs AZ4800 and AZ4126 do not impair ε‐cleavage of all investigated substrates, while the GSI L685,458 impairs ε‐cleavage of EphB2 and EphA4, but not E‐cadherin. (A) Lysates from treated BD8 cells transfected with the substrates with and without PS1 were monitored with a Luciferase reporter gene assay for ICD production using MH100 and CMV‐β‐gal constructs. The transfection efficiency was determined by β‐gal activity, and the mean value for DMSO+PS1‐treated cells was set to normalizing factor 1, and the GSI in relation to this factor. (B) Lysates from treated HEK293 cells transfected with the four substrates, MH100 and CMV‐β‐gal vectors were monitored with a Luciferase reporter gene assay for ICD production. The transfection efficiency was determined by β‐gal activity, and the mean value for DMSO‐treated cells was set to normalizing factor 1, and the GSI and GSM‐treated values in relation to this factor. (C) Western blot analysis corresponding to experiments shown in B, using a α‐VP16 antibody and β‐tubulin antibody. Bars represent a mean of four experiments (*N* = 4) in triplicates (*n* = 3) with error bars indicating SEM. The level of significance was set at *** for *p* ≤ 0.001

Several constructs of E‐cadherin, EphB2 and EphA4 were engineered in order to characterize and quantify γ‐secretase–mediated formation of both intracellular and secreted post‐proteolytic products. A well‐used and validated reporter gene strategy to quantify the release of the cytoplasmic domain in response to the first cleavage of γ‐secretase was deployed.^37^ Accordingly, a Gal4‐VP16 (GVP) transcription activation domain was inserted just C‐terminal to the membrane anchor in the cytoplasmic domain of each protein. Most of the extracellular domains of E‐cadherin, EphB2 and EphA4 were truncated in order to mimic an ectodomain‐shedded γ‐secretase substrate. The resulting constructs E‐CadΔE‐GVP, EphB2ΔE‐GVP and EphA4ΔE‐GVP are illustrated in Figure 1. First, we transfected all substrates into blastocyst‐derived stem cells, BD8 cells, which lack both presenilin 1 and 2 (PS1 and PS2), the catalytic subunit of γ‐secretase. No ICD formation was monitored in BD8 cells expressing EphB2 and EphA4, as studied by the reporter gene assay. However, co‐expression of PS1 in BD8 cells resulted in both EphB2 ICD and EphA4 ICD formation (Figure 2A). Moreover, the release of EphB2 ICD and EpA4 ICD was inhibited to more than 90% in the presence of the GSI L685,458. This inhibition is in the same range as L685,458‐mediated inhibition of AICD formation in C99‐GVP‐expressing cells (Figure 2A). These results suggest and confirm that EphB2ΔE‐GVP and EphA4ΔE‐GVP are γ‐secretase substrates (Figure 2A). In contrast, ectopic expression of E‐CadΔE‐GVP in BD8 cells resulted in significant reporter gene activity despite the lack of both PS1 and 2 expression. Moreover, neither co‐expression of PS1 or PS2 nor treatment with the GSIs L685,458, Semagacestat or DAPT impacted the amount of E‐CadΔE‐GVP ICD generated (Figure 2A and Figure S1A). Furthermore, ectopic co‐expression of C99‐GVP and PS1 in BD8 cells resulted in a similar reporter gene activity as obtained with overexpression of C99‐GVP in mouse embryonic fibroblast (MEF) cells, which express endogenous PS1. These data indicate that PS1 overexpression in BD8 cells restores γ‐secretase activity to a similar level as endogenous γ‐secretase (Figure S1B). We next transfected E‐CadΔE‐GVP, EphB2ΔE‐GVP and EphA4ΔE‐GVP into HEK293 cells, which express endogenous γ‐secretase. By using the same reporter gene assay, we could easily monitor ICD formation from each construct. However, while the processing of EphB2ΔE‐GVP, EphA4ΔE‐GVP and C99‐GVP was inhibited by the GSIs L685,458, Semagacestat and DAPT, the cleavage of E‐CadΔE‐GVP was not (Figure 2B and Figure S1C). These results were confirmed with Western blot analysis of cell lysates from the HEK293 transfectants. An antibody raised against VP16 showed a L685,458‐induced build‐up of substrate and loss of ICD formation, respectively, for C99‐GVP–, EphB2ΔE‐GVP– and EphA4ΔE‐GVP–expressing cells, while the formation of the E‐CadΔE‐GVP ICD was not affected by L685,458, which is indicated by unchanged intensity of the E‐CadΔE‐GVP and E‐CadΔE‐GVP ICD bands between the compound‐treated cells (Figure 2C). Moreover, endogenous C99 was also built up in response to L685,458 treatment, as demonstrated using an α‐AICD antibody (Figure 2D). Together, these data suggest that EphB2ΔE‐GVP and EphA4ΔE‐GVP are processed by γ‐secretase, whereas E‐CadΔE‐GVP is cleaved by other proteases.

In the next series of experiments, we used the same experimental approach to ask whether GSMs affect the amount of ICD released from EphB2ΔE‐GVP, EphA4ΔE‐GVP and E‐CadΔE‐GVP. HEK293 cells, transiently expressing either E‐CadΔE‐GVP, EphB2ΔE‐GVP or EphA4ΔE‐GVP, were exposed to three different drug‐like GSMs, AZ4800, AZ4126 and E2012, which all have been demonstrated to modulate Aβ production in vivo. Both the reporter gene and Western blot assays clearly demonstrated that the ICD formation from none of the three substrates was affected by GSM treatment (Figure 2B,C), and similar results were observed when studying the processing of endogenous C99 (Figure 2D). These data suggest that GSMs spare the initial cleavage reaction of γ‐secretase reactions.

### 3.2 Generation of Aβ‐like peptides from E‐cadherin–, EphB2‐ and EphA4‐overexpressing cells

**FIGURE 3:**
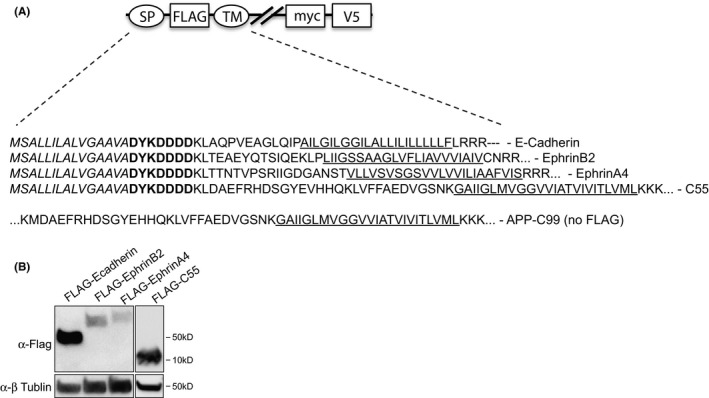
Schematic presentation of the FLAG‐tagged γ‐secretase substrates. Detected Aβ‐like peptides for FLAG‐E‐cadherin‐ΔE (FLAG‐CadΔE), FLAG‐EphB2‐ΔE (FLAG‐B2ΔE), FLAG‐EphA4‐ΔE (FLAG‐A4ΔE) and FLAG‐C55 and APP‐C99. The signal peptide is depicted in italics, and the transmembrane domain (TM) is underlined; bold amino acids represent FLAG‐tag, myc: myc‐tag, V5: V5‐tag

**FIGURE 4:**
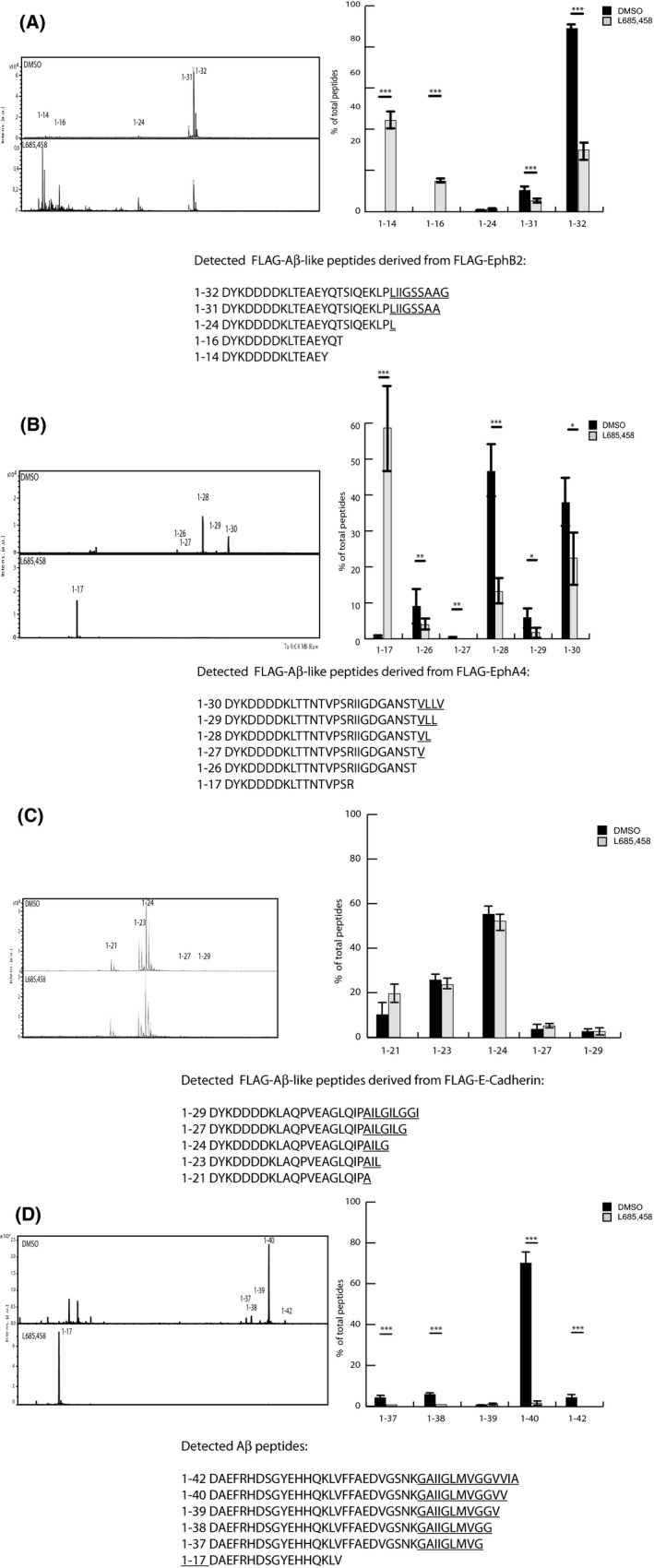
Effect of GSI on Aβ‐like peptide production. Representative MALDI‐TOF MS spectrum of secreted Aβ‐like peptides using α‐FLAG– or α‐4G8–immunoprecipitated conditioned medium from HEK293 cells stably expressing (A) FLAG‐B2ΔE, (B) FLAG‐A4ΔE, (C) FLAG‐CadΔE and (D) APPswe treated with DMSO (control) and L685,458. Each peak of the Aβ‐like peptide is plotted as a percentage of total Aβ‐like peptides. Thus, from FLAG‐B2Δ the sum of 14–32, from FLAG‐A4ΔE the sum of 17–30, from FLAG‐CadΔE the sum of 21–29 and from APPswe the sum of 37–42 are found, respectively. The bars represent the means of four to five experiments (*N* = 4–5) in duplicates (*n* = 2) with error bars indicating SEM. The level of significance was set at *p* ≤ 0.05 for *, *p* ≤ 0.005 for ** and *p* ≤ 0.001 for ***.

To further study and characterize intramembrane processing of E‐cadherin, EphB2 and EphA4, we took advantage of a combined immunoprecipitation/mass spectrometric approach to analyse peptides released into the cell culture media. N‐terminally truncated derivatives of E‐cadherin, EphB2 and EphA4 were generated, and the ECDs were replaced with a short N‐terminal FLAG‐tag in order to mimic ectodomain‐shedded proteins (i.e., to become direct substrates for γ‐secretase) and to allow a head‐to‐head immunoprecipitation and subsequent MALDI‐TOF MS analysis of secreted post‐proteolytic N‐terminal peptides from the proteins studied (Figure 3A). The resulting FLAG‐A4ΔE, Flag‐B2ΔE and FLAG‐EcadΔE constructs were stably expressed in HEK293 cells as confirmed by Western blot (Figure 3B). Analysis of the conditioned media from these cell lines revealed a different pattern of secreted N‐terminal peptides. Five peptides, ranging from 14 to 32 amino acids, were identified in conditioned media from FLAG‐B2ΔE cells (1–14, 1–16, 1–24, 1–31 and 1–32) (Figure 4A); six peptides, ranging from 17 to 30 amino acids, were identified from FLAG‐A4ΔE cells (1–17, 1–26, 1–27, 1–28, 1–29 and 1–30) (Figure 4B); and five peptides, ranging from 21 to 29 amino acids, were identified in the cell culture media of FLAG‐EcadΔE–expressing cells (1–21, 1–23, 1–24, 1–27 and 1–29) (Figure 4C). As a comparison, we could detect five different Aβ peptides, ranging from 37 to 42 amino acids in the conditioned media from HEKAPPswe cells (1–37, 1–38, 1–39, 1–40 and 1–42) (Figure 4D) and at least four different Aβ peptides (1–37, 1–38, 1–40 and 1–42) from FLAG‐C55‐expressing cells (a C‐terminal–truncated C99 substrate that retains the N‐terminal 3 amino acids of the AICD) (Figure 3 and Figure S2). While all Aβ peptides and E‐cadherin–derived peptides contained residues of the TMD region, only the FLAG‐B2ΔE–derived peptides 1–24, 1–31 and 1–32 and the FLAG‐A4ΔE–derived peptides 1–27, 1–28, 1–29, 1–30 did. The C‐terminal end of the remaining secreted FLAG‐B2ΔE and the FLAG‐A4ΔE–derived peptides originated from the extracellular juxtamembrane domain, 1–9 amino acids from the TMD.

Treatment of the cell lines with the GSI L685,458 affected the secreted peptides differently. While the generation of all secreted FLAG‐EcadΔE–derived peptides was unaffected by the GSI (Figure 4C), the level of the most shorter juxtamembrane‐derived peptides from FLAG‐EphB2 (1–14 and 1–16) and FLAG‐EphA4 (1–17) was increased. In contrast, the FLAG‐EphA4–derived peptide 1–26, which defines the entire juxtamembrane region of the EphA4 construct, was decreased by the L685,458. Similarly, all FLAG‐EphB2– and FLAG‐EphA4–derived peptides with C‐terminal ends originating from their TMDs were decreased by 50% or more by the same treatment (Figure 4A,B). All Aβ peptides derived from the FLAG‐C55 and HEKAPPswe cells were decreased by almost 100% by GSI treatment, whereas the shorter Aβ 1–17 peptide, derived from the APP juxtamembrane region, was increased (Figure 4C).

Together, these data demonstrate that the expression of FLAG‐tagged N‐terminally truncated constructs of EphA4, EphB2 and APP results in the generation of both γ‐secretase‐dependent and non‐dependent secreted Aβ and Aβ‐like peptides of varying numbers and lengths, whereas the intramembrane processing of FLAG‐E‐cadherin exclusively results in non γ‐secretase–dependent production of secreted Aβ‐like peptides.

### 3.3 GSMs show selectivity for modulation of Aβ generation

In the next series of experiments, we set out to study the impact of GSM treatment on secreted Aβ‐ and Aβ‐like peptides in the conditioned media from FLAG‐EcadΔE–, FLAG‐B2ΔE–, FLAG‐A4ΔE–, FLAG‐C55– and APPswe‐overexpressing cells.

**FIGURE 5:**
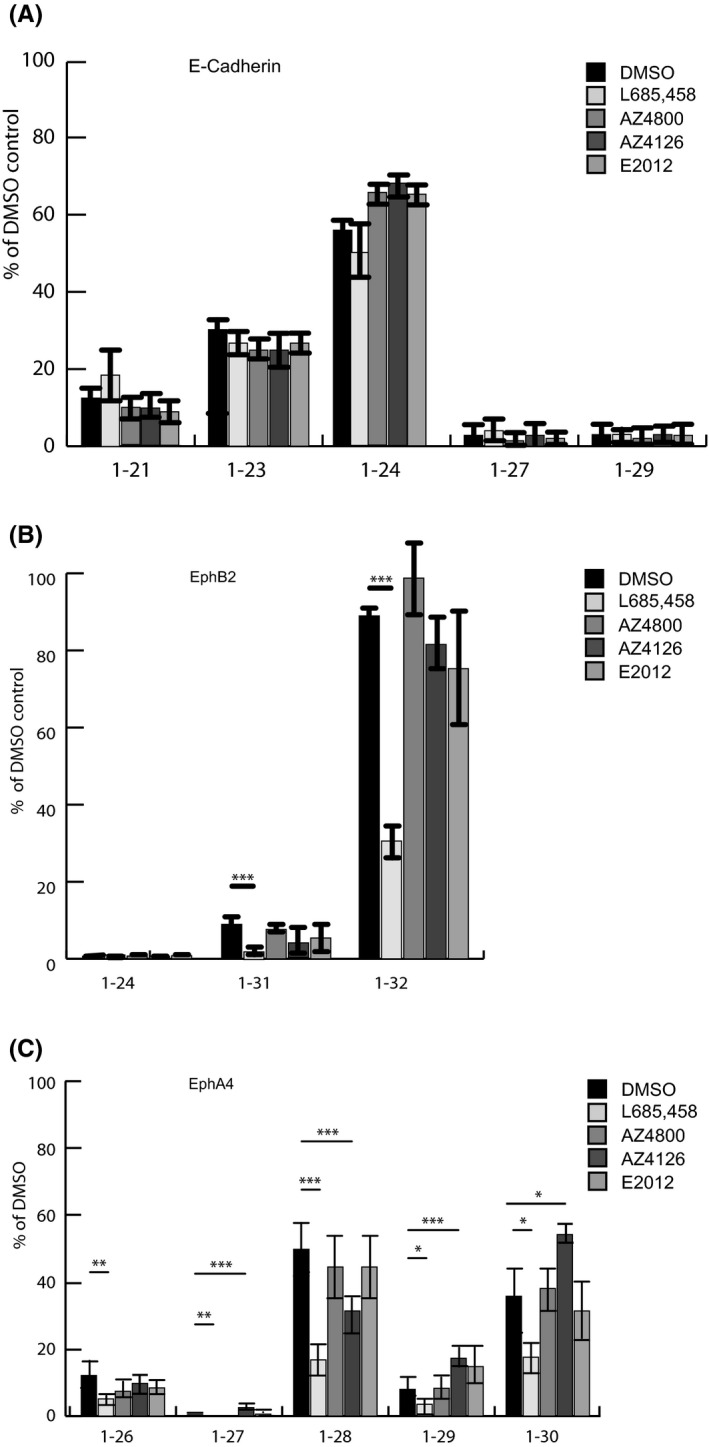
Effect of the GSMs AZ4800, AZ4126 and E2012 on Aβ‐like peptide production. Representative MALDI‐TOF MS spectrum of secreted Aβ‐like peptides using α‐FLAG–immunoprecipitated conditioned medium from HEK293 cells stably expressing (A) FLAG‐CadΔE, (B) FLAG‐B2ΔE and (C) FLAG‐A4ΔE treated with DMSO (control), L685,458, AZ4800, AZ4126 and E2012. Each peak of the Aβ‐like peptide from all substrates is plotted as a percentage of DMSO. The bars represent the means of four to five experiments (*N* = 4–5) in duplicates (*n* = 2) with error bars indicating SEM. The level of significance was set at *p* ≤ 0.05 for *, *p* ≤ 0.005 for ** and *p* ≤ 0.001 for ***

**FIGURE 6:**
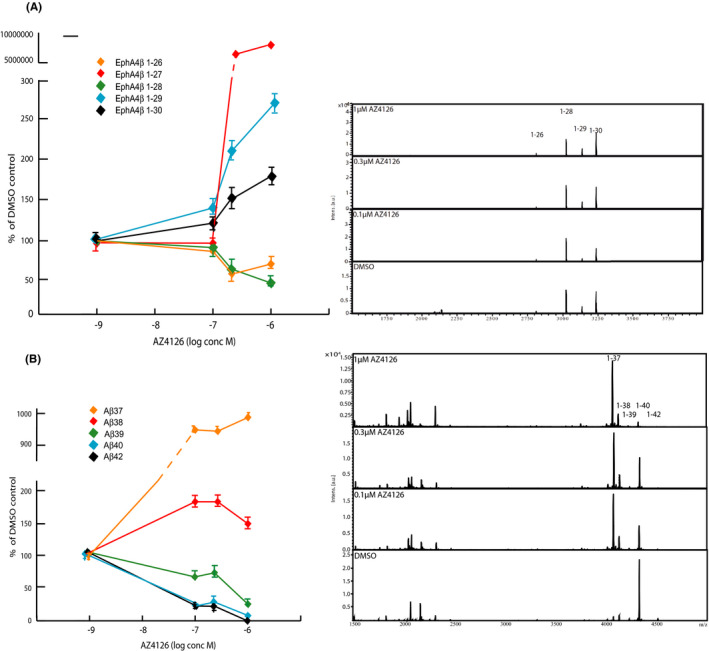
Dose‐dependent treatment with AZ4126 on FLAG‐EphA4‐ΔE and APPswe cells. (A) Schematic graph and representative MALDI‐TOF MS spectrum of showing secreted Aβ‐like peptides from HEK293 cells stably expressing FLAG‐A4ΔE treated with DMSO (control) and three concentrations of AZ4126 (1, 0.3 and 0.1 µM). (B) Schematic graph and representative MALDI‐TOF MS spectrum of showing secreted Aβ peptides from HEK293 cells stably expressing APPswe treated with DMSO (control) and three concentrations of AZ4126 (1, 0.3 and 0.1 µM). The graphs represent the means of two experiments (*N* = 2) in triplicate (*n* = 3) with error bars indicating SEM

The cells were exposed to the GSMs AZ4800, AZ4126 and E2012 for 48 h at a concentration of 400 nM for AZ4800 and AZ4126 and 1 µM for E2012, which is known to modulate the levels of secreted Aβ peptides.^5^ While the GSMs caused an expected decrease in FLAG‐extended and normal Aβ42 and Aβ40 and a concomitant increase in FLAG‐extended and normal Aβ37 and Aβ38 (Figure 6B and Figure S3), no modulation of secreted Aβ‐like peptides from FLAG‐E‐cadherin– or FLAG‐EphB2ΔE–expressing cells was monitored (Figure 5A,B). Similarly, neither AZ4800 nor E2012 modulated on the levels of Aβ‐like peptides secreted from FLAG‐EphA4ΔE–expressing cells. In fact, AZ4126 was the only GSM that affected the levels of secreted EphA4‐derived FLAG‐Aβ‐like peptides. More specifically, the 1–27, 1–29 and 1–30 peptides were increased with approximately 300%, 92% and 140%, respectively, while the 1–28 peptide was reduced with 25% (Figure 5C).

We next exposed FLAG‐EphA4ΔE‐, APPswe‐, and FLAG‐C55–expressing cells with three different concentrations of AZ4126 (0.1, 0.3 and 1 µM). While the treatment resulted in an increase in the levels of FLAG‐EphA4ΔE‐derived peptides 1–27, 1–29 and 1–30, the levels of the 1–26 and 1–28 peptides were slightly decreased, in response to 0.3 µM and 1 µM AZ4126 (Figure 6A). AZ4126 showed a more potent modulation of FLAG‐Aβ and Aβ and caused a marked decrease in FLAG‐tagged and normal Aβ40/42 and increase in Aβ37/38 in response to 0.1 µM AZ4126 (Figure 6B and Figure S2). These data suggest that the processing and release of Aβ‐like peptides derived from EphB2 and EphA4 are less susceptible to GSM‐mediated modulation as compared to γ‐secretase‐mediated APP processing.

## 4 DISCUSSION

Therapies that in a selective and tolerable manner affect Aβ42 production hold great promise as a treatment strategy in early AD. Inhibitors of both β‐ and γ‐secretases have been associated with severe safety concerns in clinical trials, and the call for alternative strategies to modulate Aβ synthesis is warranted. γ‐Secretase modulators are an emerging class of Aβ modulators that affect and prohibit amyloidogenic Aβ production, while it neither affects total APP nor Notch cleavage. While promising, a broader understanding of GSM pharmacology, beyond APP and Notch processing, is largely lacking. By combining a cellular reporter gene system of nuclear targeted proteins with mass spectrometric analysis of secreted, epitope‐tagged peptides, we have generated the experimental tools needed to study the formation of both intracellularly released and secreted post‐proteolytic products originating from the same transmembrane spanning substrate. With the use of these technologies, we provide new insights to the regulated intramembrane proteolysis of the γ‐secretase substrates EphA4, EphB2 and E‐cadherin and how these reactions are affected by several GSMs.

In order to study and characterize the formation and secretion of Aβ‐like peptides from E‐cadherin, EphA4 and EphB2, we applied an experimental strategy based on the fusion of a FLAG‐tag to the N‐terminus of constructs, which mimic ectodomain‐shedded, direct γ‐secretase substrates. In a previous study, we took advantage of this approach to characterize the processing of Notch1 and could demonstrate the secretion of the same FLAG‐extended Nβ peptides as have been shown to be generated in both the presence of an alternative N‐terminal tag and the absence of any N‐terminal modification.^10^, ^11^, ^12^, ^23^, ^24^ In this study, we have further validated the N‐terminally FLAG‐tagging approach and show that overexpression of FLAG‐tagged C55, a C‐terminally truncation of C99 lacking most of the AICD, results in a pattern of FLAG‐Aβ peptides that is similar to the Aβ profile generated from overexpressed APP. These findings are coherent with recent data from Ran et al, demonstrating that the fusion of Bri2 to either C99 or C55 results in a pattern of secreted Bri2‐Aβ peptides ending at the same C‐termini as endogenous Aβ peptides derived from the processing of APP.^38^ Together, these observations provide a rationale for the further use of N‐terminally attached FLAG‐epitope to study γ‐secretase–mediated processing of E‐cadherin, EphA4 and EphB2.

While our studies confirm that both EphA4 and EphB2 are γ‐secretase substrates, they also reveal that E‐cadherin can be processed in the absence of presenilin expression. Indeed, neither the absence of PS expression, differential PS1 or PS2 expression nor treatment with GSIs targeting both PS1 and PS2 γ‐secretases affected E‐cadherin processing in our studies. We acknowledge that our data are in sharp contrast to those published by Robakis and colleagues, which suggest that E‐cadherin is processed by γ‐secretase.^39^ At present, we do not have any mechanistic explanation to this discrepancy, but it is likely that methodological differences between the studies of Marambaud et al and ours form the basis for the different results obtained.^39^ In any case, our data clearly demonstrate that alternative metabolic pathways to γ‐secretase–mediated proteolysis exist that could mediate intramembrane processing of E‐cadherin.

Despite the difference in presenilin dependency between E‐cadherin and EphA4 and EphB2 processing, our experiments show that each of these proteins gives rise to several Aβ‐like peptides, that is secreted C‐terminally truncated peptides, upon overexpression in HEK293 cells as N‐terminally FLAG‐tagged ectodomain‐shedded proteins. The majority of these peptides have a C‐terminus that is part of the TMD of their unprocessed substrate, supporting the hypothesis that they are post‐proteolytic products of intramembrane processing.

The sensitivity of our assay allowed us to identify five secreted EphB2‐derived peptides (1–14, 1–16, 1–24, 1–31 and 1–32), six secreted EphA4‐derived peptides (1–17, 1–26, 1–27, 1–28, 1–29 and 1–30) and five secreted E‐cadherin–derived peptides (1–21, 1–23, 1–24, 1–27 and 1–29). These are relatively few peptides generated as compared to γ‐secretase–mediated APP and Notch processing, which result in the generation of eight Aβ and nine Notchβ peptides, respectively.^10^ The EphA4‐derived secreted peptides are relatively short and contain, besides the ectodomain, only 1–4 amino acids derived from the TMD. The secreted peptides derived from EphB2 extend slightly longer, 8 and 9 amino acids, respectively, into its TMD. It appears that longer peptides, such as Aβ40/42‐like species (12 and 14 amino acids, respectively, derived from APP TMD), are lacking in the repertoire of secreted EphB2 and EphA4 peptides. The molecular basis for both the difference in number and length of secreted peptides originating from different γ‐secretase–mediated reactions remains to be explained but could reflect either a limitation in the sensitivity of our assay or, alternatively, that the number of released peptides is fewer for these substrates as compared to Notch and APP. Recently, Lessard et al reported data suggesting that both the number and length of Aβ‐like peptides generated from Notch1‐4, CD44 and VEGFR1 differ. Collectively, these findings support the conclusion that the pattern and number of Aβ‐like peptides produced are highly variable and substrate‐dependent.^23^

The generation of the secreted EphB2‐derived 1–24 to 1–32 and EphA4 1–26 to 1–30 peptides was γ‐secretase–dependent. An unexpected finding was that the generation of EphA4 1–26 was inhibited by a γ‐secretase inhibitor. Indeed, the very C‐terminal T26 residue is located outside the EphA4 TMD and represents most C‐terminal amino acid of the juxtamembrane domain of EphA4. This observation implies either that the γ‐secretase processing of EphA4 occurs right at the border between the luminal site and the outer leaflet of the plasma membrane or that the ectodomain of EphA4 is recruited and moved into the active site of PS within the lipid bilayer of the membrane. Irrespective of the exact mechanism, these data suggest that γ‐secretase processing could extend beyond the amino residues normally embedded within the membrane, which, to the best of our knowledge, has never been reported before.

Besides the formation of the γ‐secretase–dependent peptides, the EphB2 1–14 and 1–16, and the EphA4 1–17 peptides were not inhibited by γ‐secretase inhibition. In contrast, these peptides were clearly elevated in response to GSI treatment. This phenomenon is reminiscent of APP processing where the levels of Aβ1‐16, derived from the APP juxtamembrane, are elevated in response to γ‐secretase inhibition as the result of α‐secretase–mediated processing of C99.^40^ It is likely that a similar situation with competing proteases is at par with EphB2 and EphA4 processing and that GSI treatment makes EphB2 and EphA4 more susceptible to cleavages by alternative proteases resulting in the increased levels of EphB2 1–14, 1–16 and EphA4 1–17, respectively.

A key objective of the current study was to study how GSM as a class of anti‐amyloid agents affects γ‐secretase–catalysed reactions in a broader context beyond APP and Notch processing. We chose to study EphB2, EphA4 and E‐cadherin processing based on their documented function and important signalling in different physiological and pathophysiological contexts, such as synaptic plasticity, memory formation, cell proliferation and cancer,^27^, ^28^, ^29^, ^30^, ^31^, ^32^, ^33^ which have been implicated in the adverse events associated with γ‐secretase inhibitor trials in AD patients.^13^, ^14^

To this end, we have studied the pharmacology of three well‐characterized GSMs: AZ4800, AZ4126 and E2012, as representative GSMs. They all belong to three chemically distinct classes of GSMs that exhibit drug‐like properties and modulate Aβ peptide production both in vitro and in vivo.^5^ Similar to Notch and APP processing, we found that none of these compounds had any effect on the formation of EphB2, EphA4 and E‐cadherin intracellular domain formation. These are promising data from a drug discovery perspective since it implicates that GSMs spare important γ‐secretase signalling beyond Notch processing and are selective for γ‐secretase.

Interestingly, despite their profound Aβ‐modulatory effect, we found that none of the GSMs modulate the production of the secreted peptides derived from EphB2 or E‐cadherin. In fact, AZ4126 was the only GSM that displayed any modulatory effect in our studies, and that on EphA4 processing. These data suggest that AZ4126 causes a dose‐dependent reduction in the shorter EphA4 1–26 and 1–28 peptides, an increase in the longer 1–29 and 1–30 peptides but also a strong elevation of 1–27 peptide, which however represents a minor part of the total peptide production. While the IC50/EC50 for AZ4126 for Aβ42/Aβ38 is 6nM, modulation of secreted EphA4 peptides was observed from 300nM AZ4126, suggesting that AZ4126 is more potent on modulating APP processing as compared to EphA4 processing and Notch processing, as we reported earlier.^10^ Together, our data therefore suggest that GSMs in general are both much more selective for APP processing and that the amplitude of modulation appears more modest on other γ‐secretase‐catalysed reactions as compared to Aβ modulation. These conclusions concur with data from Lessard and colleagues, who found that the Aβ‐like peptide formation from Notch1‐4, CD44 and VEGFR1 was not affected by GSM treatment.^23^ In addition to these findings, the authors reported that GSMs show a prevalence for PS1 γ‐secretase–catalysed Aβ production. In our study, we did not address whether the selectivity of GSMs for APP processing is affected by PS subtype–specific secretases, but it is a relevant topic for future studies.

The core pharmacology of GSMs is the decrease in Aβ40 and Aβ42 peptides and a parallel increase in shorter Aβ37, Aβ38 peptides. In that context, it is interesting to note that AZ4126 modulates EphA4 in a manner that to a large extent is opposite to APP processing and what has been demonstrated for AZ4126 on APP and Notch processing. Indeed, AZ4126 caused an increase in the longest detected EphA4‐derived Aβ‐like peptides (1–29 and 1–30) and a mix of both increased and decreased levels of shorter peptides (increase in 1–27, decrease in 1–26 and 1–28). Previous studies, which have delineated γ‐secretase–mediated APP processing and how it is affected by AZ4126, provide important insights that may explain the molecular basis of the seemingly unexpected modulation of EphA4 processing. Ihara and colleagues made the seminal finding that Aβ peptides of different lengths are generated as the result of a series of proteolytic events taking place at every 3rd or 4th residues along two major routes, denoted as the Aβ40 and 42 product lines, respectively.^7^ Accordingly, longer Aβ peptides are substrates for further γ‐secretase cleavage resulting in shorter Aβ peptides (Aβ51‐>Aβ48‐>Aβ45‐>Aβ42‐>Aβ38…, Aβ49‐>Aβ46‐>Aβ43‐>Aβ40‐>Aβ37….). Subsequent studies revealed that several additional γ‐secretase reactions take place, which results in crosstalk between the Aβ‐product lines.^5^, ^38^ In addition, Aβ40 could be a direct substrate for Aβ1‐34 and Aβ40‐35 generation, which demonstrate that γ‐secretase is not restricted to processing at every 3^rd^ or 4^th^ amino acid residue. A central take‐home message from these studies was that AZ4126 affects many γ‐secretase reactions within the product lines, crosstalk reactions and Aβ40‐35 generation.^5^ While some cleavage reactions were unchanged, several others were either increased or decreased to different degree. Thus, although the net effect of AZ4126 is an increase in secreted Aβ37/38 and a decrease in secreted Aβ40/42, the actual modulatory effect on APP processing is much more extensive and affects a larger number of γ‐secretase–catalysed processing events.^5^ To this end, we have not performed a similar analysis on EphA4 processing, but it is conceivable that AZ4126 also affects intramembrane EphA4 processing at multiple levels, which results in the mixed pattern of induced and suppressed EphA4 Aβ‐like peptides.

During the last few years, Shi and colleagues have made an incredible achievement in solving the structure of γ‐secretase and how different γ‐secretase inhibitors and modulators interact with the enzyme.^20^, ^41^, ^42^ Recently, Yang et al found that the GSM E2012 binds to the extracellular portion of γ‐secretase and may induce a clash with the N‐terminal part of the APP TMD, thereby unwinding the APP TMD N‐terminal helix.^20^ It will therefore be interesting to learn from future studies to what extent the identity of the N‐terminal portion of the TMDs of different γ‐secretase substrates dictates their differential susceptibility to GSM‐induced modulation.

In summary, we have characterized the RIP of EphA4, EphB2 and E‐cadherin and show that γ‐secretase‐mediated intramembrane processing results in the production of multiple secreted peptides from all substrates but that both the number and length of secreted peptides differ from substrate to substrate. We also demonstrate that GSMs developed for targeting Aβ production show a strong selectivity for APP processing, both in the context of intracellular domain formation and in the formation of Aβ‐like peptides. These findings are very much coherent with data from other research groups that were published during the preparation of this manuscript, and strongly support the further development of GSMs as a selective class of therapeutics targeting the Aβ amyloid component of AD.

## CONFLICT OF INTEREST

KB has served as a consultant at advisory boards or at data monitoring committees for Abcam, Axon, Biogen, JOMDD/Shimadzu, Julius Clinical, Lilly, MagQu, Novartis, Roche Diagnostics and Siemens Healthineers, and is a co‐founder of Brain Biomarker Solutions in Gothenburg AB (BBS), which is a part of the GU Ventures Incubator Program, all unrelated to the work presented in the present paper. HZ serves as a scientific consultant to Alzecure Pharma, which develops GSMs for AD. JL is a co‐founder of AlzeCure Pharma. GN is a co‐founder and employee of AlzeCure Pharma. All other authors confirm that there are no conflicts of interest.

## AUTHOR CONTRIBUTIONS

**Tobias A Weber:** Conceptualization (equal); Data curation (lead); Formal analysis (lead); Funding acquisition (supporting); Investigation (equal); Methodology (lead); Project administration (equal); Resources (supporting); Software (supporting); Supervision (supporting); Validation (lead); Visualization (equal); Writing – original draft (equal); Writing – review & editing (supporting). **Johan Lundkvist:** Conceptualization (equal); Data curation (equal); Formal analysis (equal); Funding acquisition (supporting); Investigation (equal); Methodology (equal); Project administration (equal); Resources (equal); Software (supporting); Supervision (equal); Validation (equal); Visualization (equal); Writing – original draft (equal); Writing – review & editing (equal). **Johanna Wanngren:** Data curation (equal); Formal analysis (equal); Methodology (equal); Validation (equal); Writing – original draft (equal); Writing – review & editing (equal). **Hlin Kvartsberg:** Formal analysis (equal); Methodology (equal); Writing – original draft (equal). **ShaoBo Jin:** Conceptualization (equal); Formal analysis (equal); Funding acquisition (equal); Investigation (equal). **Pia Larssen:** Formal analysis (equal); Methodology (equal); Writing – original draft (equal). **Dan Wu:** Methodology (equal); Validation (equal); Writing – original draft (equal). **Daniel V Oliveira:** Methodology (equal); Software (equal); Writing – original draft (supporting); Writing – review & editing (supporting). **Karolina Minta:** Conceptualization (equal); Formal analysis (equal); Funding acquisition (equal); Investigation (equal). **Gunnar Brinkmalm:** Conceptualization (equal); Formal analysis (equal); Funding acquisition (equal); Investigation (equal). **Henrik Zetterberg:** Data curation (equal); Supervision (equal); Validation (equal); Writing – original draft (equal); Writing – review & editing (equal). **Kaj Blennow:** Data curation (equal); Methodology (equal); Supervision (equal); Writing – review & editing (equal). **Gunnar Nordvall:** Validation (equal); Writing – original draft (equal); Writing – review & editing (equal). **Bengt Winblad:** Data curation (equal); Resources (equal); Supervision (equal); Writing – original draft (equal); Writing – review & editing (equal). **Erik Portelius:** Conceptualization (equal); Data curation (equal); Formal analysis (equal); Funding acquisition (equal); Investigation (equal); Methodology (equal); Project administration (equal); Resources (equal); Software (equal); Supervision (equal); Validation (equal); Visualization (equal); Writing – original draft (equal); Writing – review & editing (equal). **Helena Karlström:** Conceptualization (lead); Data curation (equal); Formal analysis (equal); Funding acquisition (lead); Investigation (equal); Methodology (supporting); Project administration (lead); Resources (lead); Software (equal); Supervision (lead); Validation (equal); Visualization (equal); Writing – original draft (equal); Writing – review & editing (lead).

## Supporting information

Figure S1

Figure S2

Figure S3

Table S1–S2

## Data Availability

Data available on request due to privacy/ethical restrictions.
