# Supplementary material for: γ‐Secretase modulators show selectivity for γ‐secretase–mediated amyloid precursor protein intramembrane processing
Source: J Cell Mol Med. 2021 Dec 20;26(3):880–92. doi: 10.1111/jcmm.17146 (PMC8817114; doi:10.1111/jcmm.17146)
Supplement: Supplementary file 4 — Table S1–S2 [file JCMM-26-880-s002.docx]

**cDNA and constructs**

cDNA of E-cad, EphB2, EphA4, ErbB4 was introduced in the pIRES/neo vector and p75^NTR^ was introduced in the pIRESpuro3 vector at ClaI/XmaI site and/or pcDNA3.1 vector at KpnI/XhoI site, respectively. The AscI site was introduced in the intracellular domain directly 3’ after the transmembrane region of the substrates by in vitro mutagenesis using primers (see Supplemental Table 1) according to manufacturer´s protocol (Agilent Technologies). cDNA encoding the DNA binding domain (Gal4) and transactivation domains (VP16), Gal4/VP16, (GVP) was cloned into the AscI site in each substrate, which contains the ClaI and XmaI/SmaI restriction sites when collected from the pIRES/neo vector and pIRESpuro3 vector, respectively. The FLAG-tagged constructs for E-cadherin, EphB2 and EphA4 were generated by using PCR with the primers outlined in Supplemental table 2 and the FLAG-C55 cDNA was synthezised by Genscript and cloned into pcdna3.1/Hygro (+) vector at NheI and BamHI sites with the following sequence:

gctagcgccaccATGTCTGCCCTGCTGATCCTGGCTCTGGTGGGAGCTGCTGTGGCTGACTACAAAGACGATGACGATAAGCTGGACGCTGAGTTCCGGCACGATAGCGGCTATGAAGTGCACCATCAGAAACTGGTGTTCTTTGCCGAGGATGTGGGCAGCAACAAGGGCGCTATCATTGGACTGATGGTGGGAGGAGTGGTCATCGCTACCGTGATCGTGATCACCCTGGTCATGCTGAAGAAAAAGtaatgaggatcc

Supplemental table 1.

GVP construct primers

| E-Cad_AscI_f | GCCAGCTGCACAGAGGCCGGCGCGCCAGACCCGAAGTGACCAGAAAC |
| --- | --- |
| E-Cad_AscI_r | GTTTCTGGTCACTTCGGGTCTGGCGCGCCGGCCTCTGTGCAGCTGGC |
| EphB2_AscI_f | CCCAACGAGGCCGTGCGCCGGCGCGCCAAAGAGATCGACATC |
| EphB2_AscI_r | GATGTCGATCTCTTTGGCGCGCCGGCGCACGGCCTCGTTGGG |
| EphA4_AscI_f | CCCAATCAGGCCGTGCGCCGGCGCGCCAAAGAGATCGACGCC |
| EphA4_AscI_r | GGCGTCGATCTCTTTGGCGCGCCGGCGCACGGCCTGATTGGG |

Supplemental table 2.

FLAG construct primers

| Univ_XhoI_r | CCCCCTCGAGTCACACTTTCCGCTTTTTCTTG |
| --- | --- |
| E-Cad_f | GACTACAAAGATGACGACGATAAGCTTGCCCAGCCTGTGGAAGCCGGAC |
| EphB2_f | GACTACAAAGATGACGACGATAAGCTTACAGAGGCCGAGTACCAGACC |
| EphA4_f | GACTACAAAGATGACGACGATAAGCTTACCACAAATACCGTGCCCGG |
